# Lifecycle answerability for artificial intelligence-enabled medical device software: a regulatory science perspective

**DOI:** 10.3389/fmed.2026.1896492

**Published:** 2026-08-24

**Authors:** Liu Ben

**Affiliations:** KoGuan Law School of Shanghai Jiao Tong University, Shanghai, China

**Keywords:** answerability, artificial intelligence, clinical decision support, lifecycle management, medical device software, predetermined change control plan, regulatory science, software as a medical device

## Abstract

Artificial intelligence (AI)-enabled medical device software is increasingly expected to learn, update, explain, retrieve information, draft records, and support clinical reasoning across changing care environments. Regulatory science has responded with lifecycle-oriented tools, including software-as-a-medical-device risk categorization, quality management systems, medical device software lifecycle processes, risk management, post-market monitoring, and predetermined change control plans. These tools are necessary, but they do not by themselves specify who must respond when field experience shows that an AI-supported clinical decision, workflow, validation claim, or planned update no longer fits clinical reality. Documented deployments of sepsis early-warning software in critical care—an external validation that overturned a widely deployed model’s performance claims, the manufacturer’s subsequent model replacement, and a prospective multi-site study linking alert response latency to sepsis mortality—show that such field signals are common, consequential, and unevenly answered. We propose lifecycle answerability as a regulatory science construct for AI-enabled medical device software. It specifies standing, addressee, reason-giving, temporal trigger, and revision pathway. We provisionally define the credible field signal that triggers these obligations, differentiate the construct from established algorithmic accountability frameworks, apply it in parallel to sepsis early warning and large language model documentation, and examine what is distinctive about answerability obligations in critically ill populations. Future work should test answerability through deployment case reviews, post-market signal audits, escalation pathway simulations, and implementation studies. Lifecycle answerability complements existing lifecycle governance by specifying the institutional response architecture through which credible field signals become institutionally actionable across the software lifecycle.

## Introduction

Artificial intelligence (AI)-enabled medical device software is moving from static decision support toward adaptive, reasoning, and workflow-embedded systems. Software as a Medical Device (SaMD) frameworks define intended use, clinical context, and risk (1). U.S. Food and Drug Administration (FDA) guidance on predetermined change control plans (PCCPs), and the joint FDA, Health Canada, and Medicines and Healthcare products Regulatory Agency (MHRA) guiding principles, frame planned change as a total-product-lifecycle commitment (2, 3). Quality management systems, software lifecycle processes, risk management standards, and AI risk frameworks similarly shift attention from one-time clearance to continuing control (4–7).

This movement is essential but leaves a regulatory science question under-specified: what happens when a clinical signal, patient objection, clinician dissent, subgroup performance issue, or workflow failure shows that a previously acceptable AI-supported decision or update pathway no longer fits practice? Ethics and governance frameworks identify values and call for oversight, monitoring, and clearer roles (8–12), yet value statements and lifecycle controls do not automatically create an institutional response architecture when field evidence destabilizes a clinical assumption.

We propose lifecycle answerability as a regulatory science construct for this gap. It specifies who has standing to raise a challenge, who must respond, what reasons and evidence are owed, what temporal trigger reopens a decision, and what revision pathway can change a record, workflow, model, labeling, validation claim, or PCCP. Its obligations arise, evolve, and persist across successive lifecycle stages rather than at a single decision point: the construct specifies how field evidence becomes legitimate regulatory action.

**FIGURE 1:**
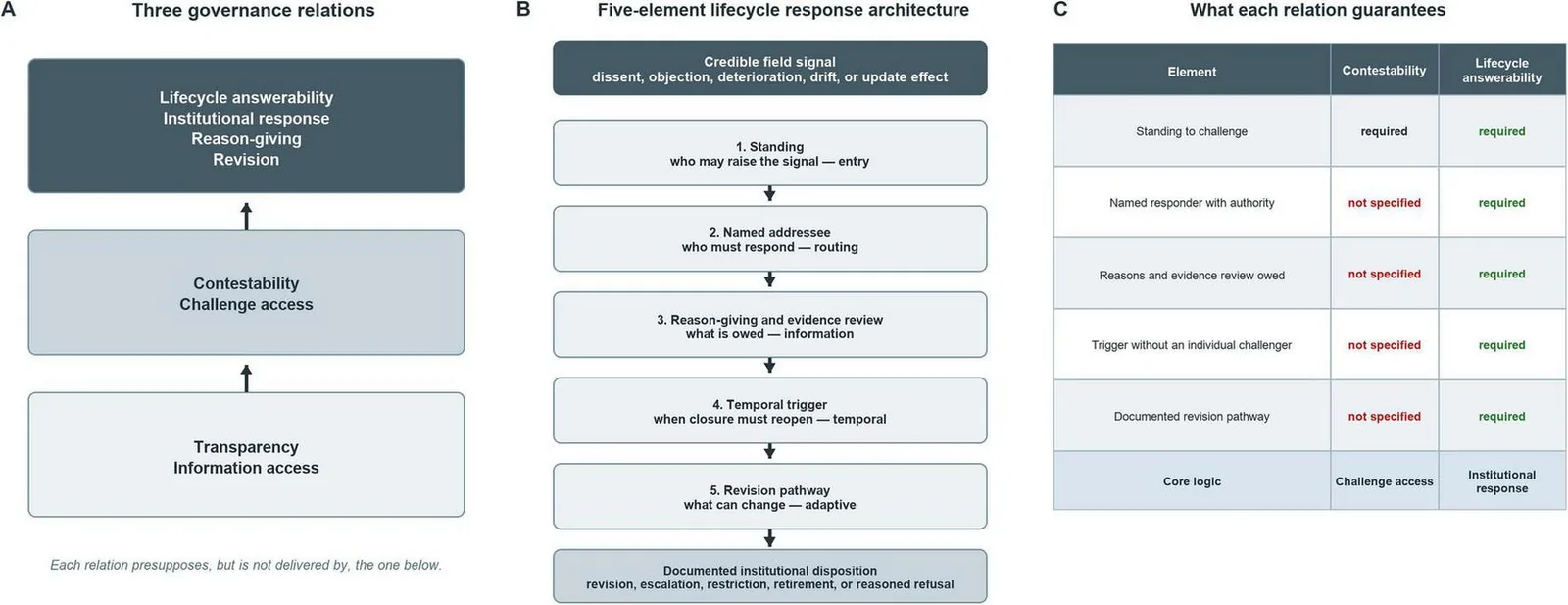
Lifecycle answerability as the institutional response architecture of lifecycle governance. **(A)** Transparency establishes what is known, contestability who may challenge, and lifecycle answerability who must respond; each relation presupposes, but is not delivered by, the one below. **(B)** The five elements perform distinct response functions: standing prevents exclusion, named addressee prevents diffusion of responsibility, reason-giving prevents unexplained closure, temporal trigger prevents governance inertia, and revision pathway prevents merely symbolic response, leading to a documented institutional disposition. **(C)** Contestability secures access to challenge, whereas lifecycle answerability additionally specifies the responder, the response owed, challenger-independent triggers, and a documented revision pathway.

The problem sharpens as AI-enabled medical device software incorporates large language models (LLMs), retrieval tools, and reasoning interfaces. Explainable AI research has long emphasized the limits of opaque models in high-stakes settings (13–17); LLMs generate fluent rationales that are not faithful accounts of the underlying computation (18, 19); and in LLM documentation, hallucinations, omissions, and clinician review are deployment rather than model-performance concerns (20). Figure 1 summarizes the construct and its relation to transparency and contestability.

## What this perspective adds

Existing lifecycle governance explains how AI changes; lifecycle answerability explains how institutions change in response to AI. The deployment record reviewed below shows why the difference matters: when field evidence contradicts prior assumptions, institutions often lack a defined response architecture. This Perspective argues that lifecycle answerability supplies that missing architecture and develops it as a five-element response architecture, translated into operational checkpoints grounded in documented critical-care deployments.

## Documented deployments: sepsis early warning in critical care

The questions this construct addresses are documented in the sepsis early-warning deployment record. The Epic Sepsis Model, a proprietary score integrated into a widely used electronic health record, was deployed at hundreds of U.S. hospitals. External validation at an academic medical center found an area under the curve of 0.63 rather than the reported 0.76–0.83: the model missed 67% of patients with sepsis while alerting on 18% of all hospitalized patients (21). The validation claim was destabilized—yet the finding surfaced through independent publication, which did not itself establish a named, time-bounded response obligation for the manufacturer or deploying hospitals. The manufacturer later replaced the model; prospective multicenter validation of the update reported better discrimination but marked between-institution variability, low positive predictive value, and high alert burden, recommending local validation (22). The update thus generated new deployment-side obligations: local revalidation, threshold re-derivation, and reinterpretation of prior records.

The Targeted Real-time Early Warning System (TREWS) shows the response side of the same architecture. In a prospective multi-site study of 590,736 monitored patients, sepsis alerts confirmed by a provider within three hours were associated with a 3.3% absolute (18.7% relative) reduction in in-hospital mortality (23), with adoption and timing effects varying across providers (24), and qualitative work documenting how clinicians trusted, dismissed, or escalated alerts (25). Response latency, dismissal patterns, and confirmation rates are thus measurable, mortality-relevant institutional variables—the routing and temporal dimensions that lifecycle answerability names.

Consider a patient who deteriorates into septic shock after a non-alert and later says: my diagnosis was delayed. Lodging and recording the complaint satisfies the entry condition of contestability; lifecycle answerability asks what institutional response follows. Who receives the challenge: the clinician, the hospital’s governance body, or the manufacturer? Who owes an explanation of why no alert fired, on what evidence: local validation, subgroup performance, threshold rationale, override history? Can the threshold be reopened, by whom, and does revision fall within the authorized PCCP or require a new submission path? And who corrects the record so that the delayed diagnosis does not silently shape future care? Authority was distributed across several actors, but the published record identifies no mechanism guaranteeing a named, time-bounded response. These deployments thus expose a common institutional problem: field signals emerge, yet no response architecture specifies who must answer, by when, or through which revision pathway. The remainder of this Perspective develops that architecture.

## What is distinctive in critical illness and infectious disease

Four features sharpen answerability obligations in critically ill and septic populations. First, decision windows are narrow: hourly delays in sepsis bundle completion and antibiotic administration are associated with increased mortality (26), so response latency, not only occurrence, is a patient-safety parameter measurable via the TREWS 3-h window (23). Second, contestation capacity is constrained: patients in septic shock or under sedation cannot challenge outputs, and informed consent is often impracticable; standing must therefore extend to surrogates and retrospective review, and challenges typically arrive after harm. Third, alert density is high: when a model alerts on nearly one fifth of hospitalized patients (21), alert fatigue converts drift into silent failure and overrides become ambiguous signals that only institutional review can interpret. Fourth, error costs are asymmetric: missed alerts delay antibiotics for the indexed patient, while false alerts drive unnecessary broad-spectrum antimicrobial exposure, with stewardship and resistance costs beyond the individual encounter.

## Why lifecycle answerability is needed

The deployment record illustrates three connected problems. First, closure is necessary for action, but premature or institutionally rigid closure makes later alternatives costly: premature diagnostic closure contributes to diagnostic error, and automation bias and verification complexity reduce vigilance (27–30); the same pressure appears in validation claims, workflow defaults, and planned update pathways. Second, explanation does not stabilize meaning: plans, records, and model outputs are interpreted in practical settings and permit multiple local readings (31–34); AI explanations show mixed effects on performance; and healthcare explainability remains social and institutional rather than merely technical (35–37). Third, clinical care is revisable: shared decision-making research treats decisions as constructed and revisited, with AI reshaping who can reopen them (38–43), and ethnographic and naturalistic research describes treatment as continuous adjustment and expert judgment as cycles of assessment and revision (44–48). When field evidence destabilizes a model output, workflow, validation claim, or planned change, the system should specify who can raise the concern, who must respond, and what can change. These problems persist even where contestation is available.

## Why lifecycle answerability is not equivalent to contestability

Contestability addresses only the entry point. Contestable AI by design keeps decisions open to dispute, letting individuals and communities contest outputs, data, models, and design choices (49). Answerability asks which institution must respond once a challenge—or a trigger arising without any challenger—arrives (Figure 1A). The two relations diverge at four points (Figures 1B, C): answerability additionally requires a named addressee with authority over the challenged element; an owed response of reasons and evidence review, so that receipt without response is a documented failure; temporal triggers (drift, subgroup change, update effects) that operate without any challenger; and a defined revision pathway whose outcome, including reasoned refusal, is recorded. Contestability determines whether communication can begin; answerability, whether it produces institutional change.

The sepsis deployments show the divergence in both directions. TREWS alert dismissals were recordable instances of clinician-system disagreement, but they became institutionally consequential only where their patterns were measured and reviewed (23, 25). Conversely, the Epic model replacement did not depend on an individual contestation event—contestability was silent—yet it obligated deployers to revalidate locally and answer for how records created under the prior model should be read (22). A system can be fully contestable yet not answerable, and answerability obligations can arise without any contestation event (Figure 1C).

## Lifecycle answerability as a regulatory science construct

Lifecycle answerability is the institutional response architecture through which credible field signals become assigned response obligations, evidence review, and documented disposition across the AI-enabled medical device software lifecycle. It is narrower than accountability and broader than transparency: it asks whether a challenge raised by a legitimate actor reaches an addressee with authority to give reasons, evaluate evidence, and initiate proportionate revision (50–52).

Five elements follow, each addressing a distinct institutional failure: standing (who may raise a challenge) prevents exclusion of legitimate challengers; addressee (who must respond) prevents diffusion of responsibility; reason-giving (what evidence, rationale, uncertainty, or audit trail is owed) prevents unexplained institutional closure; temporal trigger (when closure must be reopened) prevents governance inertia; and revision pathway (what can change, from record and care plan to threshold, labeling, validation claim, monitoring plan, PCCP, or retirement) prevents merely symbolic response. Answerability is bounded by role, evidence threshold, proportionality, clinical urgency, and patient safety: decisions close so that action can occur; the question is whether closure remains revisable.

## What makes a field signal credible

The trigger—a credible field signal—needs provisional content. A signal is credible when it meets criteria fixed before deployment, of three kinds: categorical single-case signals, qualifying without statistics on sentinel-event logic (deterioration to septic shock after a non-alert, with documented harm); statistical pattern signals, qualifying when monitored indicators—override rates, subgroup positive predictive value, calibration—cross pre-specified control limits, distinguishing an isolated complaint from a pattern of drift; and material update signals, qualifying automatically when a manufacturer change may affect intended use, validated performance, or clinical interpretation, as with the Epic replacement (22). These criteria are illustrative rather than exhaustive. Screening is itself answerable: the designated addressee must document reasons for acting or not within a defined interval. Because criteria are ex ante and dismissals are recorded, credibility does not reduce to whatever the institution happens to answer; the definition is not circular.

## Relation to algorithmic accountability frameworks

The construct builds on accountability scholarship. Bovens defines accountability as an actor’s obligation to explain conduct to a forum that may question, judge, and impose consequences (53); lifecycle answerability institutionalizes that relation inside the device lifecycle, except that the accountable actor is distributed—manufacturer, deployer, and regulator hold different obligations for the same signal—and the forum’s episodic demand is replaced by pre-specified triggers that operate even when no forum convenes. It borrows the structure, not the subject, of Shoemaker’s answerability (50): the system that produced the output cannot answer for it, so obligations attach to institutional roles. Kroll and colleagues develop ex ante technical assurance designed into systems (54); answerability is the post-deployment complement. The closest neighbor, the internal-audit framework of Raji and colleagues, assigns responsibilities across the development lifecycle (55); but audits are organization-initiated and periodic where answerability is signal-triggered and continuous, standing extends to patients and clinicians owed a response, and an audit is one revision pathway among several into which signals are routed. Diakopoulos’s accountability reporting supplies external challenge without institutional response obligation (56), precisely the asymmetry the construct is designed to address.

## Why existing corrective action is necessary but not sufficient

Post-market surveillance and corrective and preventive action (CAPA) supply part of this architecture. FDA’s Quality Management System Regulation, which since February 2, 2026 incorporates ISO 13485:2016 by reference (4, 57), requires corrective action to include cause analysis and review of the effectiveness of the action taken (clause 8.5.2), replacing—while functionally continuing—the former 21 CFR §820.100 requirement to verify or validate that corrective action is effective (58); complaint investigation and device-performance surveillance are likewise addressed (59). CAPA thus already institutionalizes owed follow-through and evidence review within the manufacturer’s quality system; the gap identified here must be stated precisely, not absolutely.

What CAPA does not supply is captured by three differences. Standing: complaint and corrective-action processes investigate signals, but the effectiveness review is owed to the quality system and its auditors, not to the patient or clinician who raised the concern; receipt without a reasoned response to the challenger is not a CAPA failure. Scope: several revision pathways in the sepsis deployments—escalation protocols, local thresholds, reinterpretation of prior records—belong to the deploying organization and fall outside a manufacturer-only process; the Epic model’s local validation shortfalls were not manufacturer nonconformities at all (21). Allocation: no mechanism specifies which addressee among manufacturer, deployer, clinical team, and regulator owes a response to each signal class. Answerability may arise before, or independently of, the manufacturer’s threshold for corrective action.

The same asymmetry runs through other lifecycle mechanisms: SaMD risk categorization confers no standing to contest local fit as use drifts (1); software lifecycle processes can close requirements before clinical interpretation stabilizes (5); risk files may not reopen after deployment (6); the NIST AI RMF does not allocate these response relations (7); and PCCPs may not anticipate field-triggered concerns (2). What is missing in each case is not a control but a response architecture. Lifecycle answerability therefore operates as a coordination layer across existing mechanisms rather than as an additional one: CAPA and its neighbors are revision pathways among which the construct routes each credible field signal.

## Operational implications for AI-enabled medical device software

Table 1 translates the construct into regulatory and clinical phases and names, for each phase, the addressee owing the first response. A field signal should not be classified as a user, model, quality, or regulatory problem before the addressee is clear. Signals arise after deployment, but revision pathways may reopen earlier stages, including problem formulation and validation.

**TABLE 1:** Lifecycle answerability matrix for AI-enabled medical device software, with the responsible addressee named for each lifecycle phase.

Lifecycle phase	Closure risk	Answerability trigger	Responsible addressee	Possible revision
Problem formulation	Clinical problem definition becomes frozen.	Patient, clinician, or community contests intended use or excluded use.	Manufacturer, with regulator review	Revise problem framing, use limits, stakeholder requirements, or labeling.
Data/model development	Data gaps become hidden authority.	Bias, missingness, subgroup uncertainty, or representativeness concern appears.	Manufacturer (development and quality teams)	Revise data documentation, model objective, bias response plan, or evidence claim.
Validation	Validation is treated as final proof.	Local fit, residual uncertainty, or external validity is challenged.	Manufacturer and deploying organization	Set revalidation thresholds, site-specific validation, or deployment limits.
Regulatory/PCCP	Planned change can become overly narrow.	Clinical signal falls outside the planned modification pathway.	Manufacturer and regulatory authority	Escalate to safety review, labeling change, PCCP revision, or new submission path.
Deployment	Output becomes workflow default.	Overrides, handoff failures, alert fatigue, or role ambiguity recur.	Deploying organization (clinical governance)	Revise escalation protocol, record design, training, or role responsibilities.
Clinical use	Contestation is treated as friction.	Clinician dissent, patient objection, or deterioration contradicts the output.	Clinician and local governance body	Revise patient record, care plan, explanation, threshold, or escalation route.
Monitoring	Drift signals remain technical.	Performance degradation or subgroup harm emerges.	Manufacturer quality unit with deploying organization	Trigger audit, local recalibration, broader post-market action, or restriction.
Update/retirement	Residual effects persist after change or withdrawal.	Updated or retired system affects prior records, decisions, or workflows.	Manufacturer, deploying organization, and regulator	Notify users, correct records, reinterpret affected decisions, or retire tool.

The construct applies with equal specificity to LLM-enabled documentation. Ambient AI scribes, deployed at health-system scale rather than in critical care specifically, exceeded 10,000 physicians and 2.5 million uses in one system within a year (60, 61), with hallucinations and omissions as central safety concerns (20). Standing: the patient who finds a fabricated finding, the clinician asked to sign generated text, and records staff detecting systematic errors. Addressee: the deployer’s documentation governance owns record content and workflow; the manufacturer owns model version, speech pipeline, and configuration. Reason-giving: provenance is owed—which text derives from transcript versus generation, under which model and configuration version, with which known error classes. Temporal triggers: sampled hallucination and omission rates crossing control limits, manufacturer model updates, and scope creep into specialties or encounter types absent from validation; at millions of uses review is necessarily sampling-based, so monitoring design is itself an answerability object. Revision pathways: correction of the record and downstream documents that inherited the error, configuration rollback, use-case restriction, or retirement. Many scribes sit outside device regulation; these obligations attach to the deploying institution regardless—one reason the construct is framed institutionally and sociotechnically rather than only regulatorily (62, 63).

## Discussion: implications and future directions

First, researchers should measure lifecycle closure pressure: how quickly a system settles uncertainty, how costly reopening is, and whether disconfirming information is still sought after an AI output appears.

Second, manufacturers and deploying organizations should build interpretation-preserving infrastructure. Uncertainty records, dissent capture, versioned decision trails, local validation thresholds, and revalidation pathways should be treated as linked parts of lifecycle control, so that a clinical concern can change what is monitored, validated, updated, or retired.

Third, regulators should ask how field-triggered concerns interact with planned-change mechanisms. The joint FDA, Health Canada, and MHRA guiding principles frame robust PCCPs as focused, risk-based, evidence-based, transparent, and lifecycle-oriented (3), but answerability asks what happens when clinical reality reveals an unplanned concern or the planned pathway no longer fits.

Fourth, the framework is testable: richer response architectures should show shorter response latency, more transparent revision, and higher adaptive capacity. Future work can operationalize these as measurable variables—routing latency, response completeness, revision traceability—through deployment case reviews, post-market signal audits, escalation-pathway simulations, and implementation studies. The deployments provide reference cases: the interval between external invalidation of the Epic model and local threshold action, and the distribution of TREWS confirmation latencies (21, 23). Existing frameworks allocate responsibilities across lifecycle stages; lifecycle answerability allocates institutional response after deployment. If predictive performance measures whether AI makes better decisions, lifecycle answerability measures whether institutions remain capable of correcting those decisions after deployment.

## Limitations and boundary conditions

Lifecycle answerability is a conceptual construct that awaits empirical validation, and the patient-level vignette above is an illustrative composite anchored to documented findings, not a reported case. Thresholds will vary across jurisdictions, device risk, and organizational capacity. Implementation carries its own risk: thresholds set too low create documentation burden; set too high, early drift or workflow misfit may never become institutionally actionable.

Two further limitations warrant emphasis. First, the construct does not yet formalize signal transfer across addressees. When a complaint is received by a clinician but its cause lies upstream in model validation or subgroup performance, the five elements specify that routing and response are owed, but not how substantive responsibility apportions between deployer and manufacturer. Formalizing transfer rules—joint root-cause obligations, time-bounded handoffs—is a future research direction.

Second, implementation capacity is unevenly distributed. The architecture presumes governance committees, monitoring infrastructure, and informatics staff that well-resourced academic systems possess and that many community hospitals and low- and middle-income-country health systems do not (64). In global infectious disease contexts, an unfunded mandate could concentrate safe AI deployment in resource-rich settings. Mitigations include proportionality to risk class, manufacturer-side signal intake and subgroup monitoring to reduce deployer burden, and shared or regional governance. An architecture only wealthy institutions can afford would reproduce the disparities it is meant to detect.

## Conclusion

AI may appropriately support clinical decisions when safe, validated, and contextually governed. The further issue is whether decisions, models, workflows, and regulatory claims remain answerable after closure becomes unstable—a condition the sepsis record shows is common rather than exceptional. Lifecycle answerability complements existing governance by specifying the institutional response architecture through which credible field signals remain capable of changing decisions, workflows, and regulatory claims after deployment. In this sense, lifecycle answerability extends lifecycle governance from managing software evolution to managing institutional adaptation.

## Data Availability

The original contributions presented in this study are included in this article/supplementary material, further inquiries can be directed to the corresponding author.
